# Increasing efficiency and well-being? a systematic review of the empirical claims of the double-benefit argument in socially assistive devices

**DOI:** 10.1186/s12910-023-00984-z

**Published:** 2023-11-30

**Authors:** Joschka Haltaufderheide, Annika Lucht, Christoph Strünck, Jochen Vollmann

**Affiliations:** 1 Medical Ethics with a Focus on Digitization, Joint Faculty for Health Sciences Brandenburg, University of Potsdam, Am Mühlenberg 9, 14476 Potsdam, Germany; 2 Institute for Medical Ethics and History of Medicine, Ruhr-University Bochum, Bochum, Germany; 3 School of Life Sciences, University of Siegen, Siegen, Germany; 4 Institute of Gerontology at Technical University Dortmund, Dortmund, Germany

**Keywords:** Health care technology, Health services for the aged, Medical ethics, Systematic review, Socially assistive devices, Care robots, Autonomy, Well-being

## Abstract

**Background:**

Socially assistive devices (care robots, companions, smart screen assistants) have been advocated as a promising tool in elderly care in Western healthcare systems. Ethical debates indicate various challenges. One of the most prevalent arguments in the debate is the double-benefit argument claiming that socially assistive devices may not only provide benefits for autonomy and well-being of their users but might also be more efficient than other caring practices and might help to mitigate scarce resources in healthcare. Against this background, we used a subset of comparative empirical studies from a comprehensive systematic review on effects and perceptions of human-machine interaction with socially assistive devices to gather and appraise all available evidence supporting this argument from the empirical side.

**Methods:**

Electronic databases and additional sources were queried using a comprehensive search strategy which generated 9851 records. Studies were screened independently by two authors. Methodological quality of studies was assessed. For 39 reports using a comparative study design, a narrative synthesis was performed.

**Results:**

The data shows positive evidential support to claim that some socially assistive devices (Paro) might be able to contribute to the well-being and autonomy of their users. However, results also indicate that these positive findings may be heavily dependent on the context of use and the population. In addition, we found evidence that socially assistive devices can have negative effects on certain populations. Evidence regarding the claim of efficiency is scarce. Existing results indicate that socially assistive devices can be more effective than standard of care but are far less effective than plush toys or placebo devices.

**Discussion:**

We suggest using the double-benefit argument with great caution as it is not supported by the currently available evidence. The occurrence of potentially negative effects of socially assistive devices requires more research and indicates a more complex ethical calculus than suggested by the double-benefit argument.

**Supplementary Information:**

The online version contains supplementary material available at 10.1186/s12910-023-00984-z.

## Background

Socially assistive technologies (SATs) have been gaining popularity in healthcare as a means of providing care and support, to increase or maintain well-being and autonomy, and to allow for societal participation despite limitations [1–3]. This is particularly true with regard to care for the elderly, where SATs are increasingly being used. These technologies, which include care robots, smart screen assistants, virtual avatars, and companion devices can be characterized by entailing at least three essential features. Firstly, they integrate into the everyday lifeworld of their users [4, 5]. Secondly, they offer support by taking over control, routine, or steering tasks or by acting with or on behalf of their users [1, 4, 5]. Finally and most importantly, SATs provide their services through a special interface that resembles interacting with an animate being [6–9]. This can include, for example, anthropomorphic or zoomorphic designs, mimicking social behavior, the display of emotional states, personalities, wishes and desires, or communication through natural language interactions [10]. SATs use complex digital technologies to detect actions or reactions of their human counterpart including face or gesture recognition or emotion and language models to react accordingly. This results in interaction patterns that resemble more intuitive ways of human communication. It often allows for easy access to complex supportive services or enriches human-machine interaction with an emotional or social dimension [9, 11, 12].

Connected to this concept are, however, a variety of ethical questions [13]. On the one hand, arguments highlight a tailored fit between services provided and elderly people’s needs [14, 15]. They emphasize the ethical importance of autonomy, individual freedom, and social participation [13]. On the other hand, critical voices claim that SATs challenge long-standing caring practices [16, 17] based on arguments of efficiency, sacrificing the value of human contact over a technical rationalization of care processes [18–20]. In addition, the social interface of SATs has raised concerns regarding the possible infantilization of users [21], their probable deception [22–24] or a loss of autonomy due to SATs deeply integrating into everyday life and silently winning control as technical background pacemakers.

One of the most commonly found arguments within these debates is the double-benefit argument. It claims that SATs do not only provide opportunities to increase well-being and autonomy for individual users. In addition, they may provide a systemic benefit for healthcare by mitigating scarce resources, for example, by taking over routine tasks or by relieving human care workers of burdensome tasks and, thus, creating opportunities to be more concerned with high-quality care. Given that its basic claims refer to the well-being and autonomy of users it is an argument with essentially ethical underpinnings.

If one accepts the double-benefit argument as conclusive and sound, it provides a strong basis to justify addressing research gaps, to argue for a broader implementation and use from an ethical perspective or to argue for the plausibility of certain future scenarios which include the use of SATs. However, its validity does not only rest on ethical assumptions about the value of autonomy and well-being. As a so-called mixed moral judgment, it is also reliant on the plausibility of several empirical claims [25]. Whether these empirical assumptions hold has scarcely, if ever, been considered in ethical literature. Against this background, we conducted a comprehensive systematic review gathering all relevant empirical data on the use of SATs and user experiences in elderly care to evaluate and critically appraise the empirical assumptions of the double-benefit argument. As these empirical claims are of comparative type, we used the subset of comparative studies and specifically analyzed the available data with regard to the question of whether, to what extent, and for what devices the empirical premises of the argument can be verified or made plausible by existing empirical research. In what follows, we will first, reconstruct a brief outline of the double benefit argument to familiarize the reader with its structure and to identify its empirical claims. We will, then, briefly outline the methods for conducting the review, including the search, screening of the records and synthesizing of evidence. We will, then, present our findings with regard to our research question and discuss their implications.

### A brief outline of the double-benefit argument

One of the most prevalent ethical arguments in debates surrounding research, implementation and use of SATs in elderly care is the double-benefit argument. It is commonly made or referenced in various versions and different types of research. For example, in the published dataset of a recent systematic review of Vandemeulebroucke et al. [13] we found that 10 out of 28 included articles investigating the ethical issues of social robotics in elderly care considered their arguments against the background of the double-benefit, or highlighted or referenced various versions [18–21, 26–31].

For the purpose of this paper, we will reconstruct the general structure of the argument with the aim of identifying its supportive empirical claims. We will not provide a complete logical reconstruction as this, firstly, would be well beyond the scope of this paper. Secondly, we do not aim to investigate the argument’s logical validity and soundness. With this in mind, we suggest understanding the double-benefit argument as a series of so-called mixed moral judgments [25] which combine statements about normative claims with statements about empirical observations to conclude what ought to be done or to develop ethical recommendations. To our understanding, the argument proceeds in three steps presupposing the validity of at least two normative premises which entail that (a) autonomy and well-being of care recipients ought to be protected and (b) that this extends to foreseeable harm to care recipients in the future which ought to be prevented.

In the first step, it is argued that especially western healthcare systems will face enormous challenges in the near future. Due to demographic factors, a growing number of increasingly older persons will be reliant on care and health support. Due to a prolonged life expectancy and increasing motoric, sensory or cognitive limitations, these persons will also need care for a longer time. On the other hand, however, social structures providing the necessary resources are changing. Factors such as increasing female labor force or higher mobility together with a relatively decreasing share of younger persons will lead to an overall decrease in persons being able to provide care and support or to generate the necessary resources. It is, hence, assumed to be likely that the resources of current healthcare systems will not suffice to satisfy all morally justified needs in the future. Given a moral obligation to protect justified claims of care recipients and to prevent forseeable harm in the future, it can be concluded that healthcare should be transformed to prevent to occurrence of future harm as a result of a foreseeable scarcity of resources.

While step one develops a problem-oriented perspective that argues for the adoption of certain morally valuable ends to comply with the requirement of preventing future harms, the second step establishes adequacy of means by claiming that digital healthcare technologies may contribute to the necessary changes as they provide opportunities to increase efficiency in caring practices and, hence, mitigate scarce resources. With regard to SATs, it has, for example, been argued that these devices provide opportunities to relieve caregivers from burdensome or repetitive tasks or to allow them to engage in high-quality care. SATs, hence, would provide a viable alternative to support or supplement existing caring practices while consuming fewer resources. Given the above-named moral obligations, this allows to conclude that SATs provide a more efficient way of satisfying moral requirements. This is preferable under scarce resources.

Thirdly, it is argued that such increased efficiency will not come at the expense of individual users’ autonomy or well-being. In this regard, it is often suggested that SATs can provide a tailored fit to the needs of elderly care receivers, allowing them to maintain their agential capacities or even to increase autonomy and well-being despite growing limitations. Against the background of the normative claim that autonomy and well-being ought to be protected, it can be concluded that SATs do not only provide a means to prevent foreseeable harm in the future but also do not harm present users and might even provide additional benefits. Using additional and non-trivial premises, it can, for example, be argued that a measure that contributes to the transformation of healthcare while not harming anyone and at least benefiting some of its users should be broadly considered, furthered through additional research or implemented [32].

Especially the second and third step of the argument, the ones we will be concerned with in this article, have raised several objections and criticism referring to their rhetoric, strategic or conceptual underpinnings. Sparrow has, for example, argued that these claims are often brought forward in a hyperbolic way to justify certain research or research interests [20], while others have noted a tendency in the debate to overstate the potential of care technologies. Further objections can be raised with regard to the concepts of autonomy and well-being (of users) which can be criticized as either being too narrow, to exclude other important stakeholders (for example caregivers) or value perspectives (for example the value of care) [33, 34]. However, the empirical presuppositions made with the double-benefit argument have received less attention in the literature. With this in mind, we identify two major claims in steps two and three that seem to bear most of the argumentative load on this side:

1. The efficiency claim, that is, SATs provide a more efficient means to the end of transforming the healthcare system compared to usual care practices or existing alternatives.
2. The individual well-being claim, that is, SATs do not harm current users or might even provide additional benefits in regard to increased well-being and autonomy.

The question of our analysis was, hence, whether and to what extent these claims find support in existing empirical research.

## Methods

To answer this question, we used the subset of comparative studies from a comprehensive review of empirical evidence on human-machine interaction with SATs in healthcare. The results from the arm of non-comparative studies were reported elsewhere [5]. The review protocol was designed and agreed upon by the authors. It was subsequently registered in the prospective register of systematic reviews (CRD42020160853). The aim was to gather all available empirical studies concerned with the effects and perceptions of human-machine interaction with SATs in healthcare. Screened articles were included based on a set of operationalized inclusion and exclusion criteria defined by population, devices and healthcare settings. Finally, a narrative synthesis was conducted.

### Inclusion and exclusion criteria

We determined that the focus of our review should be on the typical use settings of SATs. This includes everyday use in typical care and support settings such as homecare, nursing homes, geriatric care settings and rehabilitation settings. To determine whether a device qualifies as socially assistive technology, we used the definition outlined above which was derived from the literature. With regard to the outcomes, we included all studies that explored or investigated the effects on users of SATs. The population criterion was operationalized by determining that at least half of the study population should be above 18 years and display some kind of need for health or care support. This criterion was added to avoid including types of studies that are usually conducted in the technical sciences and which predominantly include mock populations to test the feasibility or usability of devices. We hypothesized that the results of such studies would not be comparable to real-life settings. The population criterion was checked using demographic information reported in the studies. We also included studies that reported on additional groups such as caretakers or relatives. In this case, only relevant data were extracted.

Theoretical articles, editorials, study protocols and so-called wizard-of-oz studies were excluded. In the latter, the device is usually remote-operated by a person and, hence, does not present a case of interaction with SATs as outlined above. This also applies to studies with devices whose whole purpose is to connect to other persons (e.g. via video calls). In addition, single case studies and proof-of-concept studies were also excluded.

### Search

**Fig. 1:**
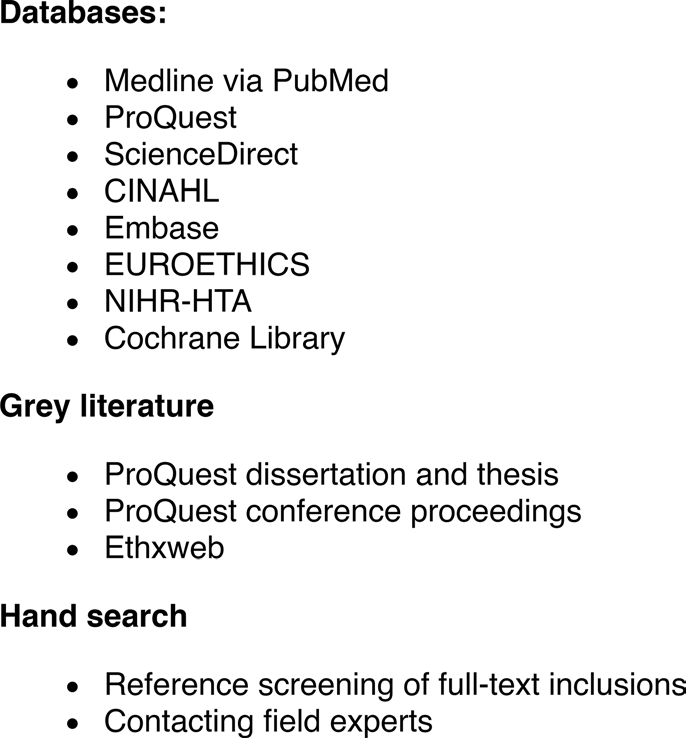
Sources for conducting the search

Database searches were carried out in February 2020 with an update in May 2021. Databases included were Medline via PubMed, ProQuest, ScienceDirect, CINAHL, Embase, EUROETHICS, NIHR-HTA and Cochrane Library. In addition, we searched for grey literature, citations of full-text inclusions, scanned conference proceedings and consulted with experts from the field. Detailed information on the sources can be found in Fig. 1. Details on the search strategy can be found with the protocol.

### Selection and data extraction

Three of the authors screened titles and abstracts with the help of two assistants who were supervised by the first author. The assistants’ decisions were separately reviewed. Each record was screened twice. Full texts were screened independently by two authors. In case of disagreement in the stage of title and abstract screening, the final decision was postponed until the full text was accessed. In case of disagreement within the full-text stage, a third author was consulted. The data was extracted by two authors independently using a modified data collection form based on the data collection template of the Cochrane Foundation. Subsequently, both author extractions were checked against each other to avoid loss of information. Reasons for exclusions were documented in all stages. In case of missing or unclear information or where reports indicated the existence of additional publications, the study authors were contacted.

### Assessment of methodological quality

Although not planned in advance and with the protocol, we decided to appraise methodological quality using the MMAT tool for mixed-methods reviews [35]. This decision was made against the background of a very diverse study landscape and to be able to present a better overview of all studies. The appraisal was conducted independently by two authors and was, then, synthesized. Disagreements were resolved during discussion.

### Synthesis

A synthesis was performed following Pope et al. [36]. As this approach is meta-aggregative in nature, we hypothesized that this would allow us to develop a synthesis out of the diverse study base. For this analysis, we chose preliminary core categories in line with our research questions, which were then inductively refined by aggregating material. For this purpose, we adopted a broad view on autonomy and well-being, defining the first as the ability for self-determination and well-being as a necessary and immediate prerequisite to set goals and to be able to pursue them through one’s actions. The authors, first, independently compiled findings which were, then, synthesized in a joint coding using MaxQDA as software. Reporting follows PRISMA-Guidelines where applicable. The PRISMA checklist can be found in Additional file 1.

## Results

**Fig. 2:**
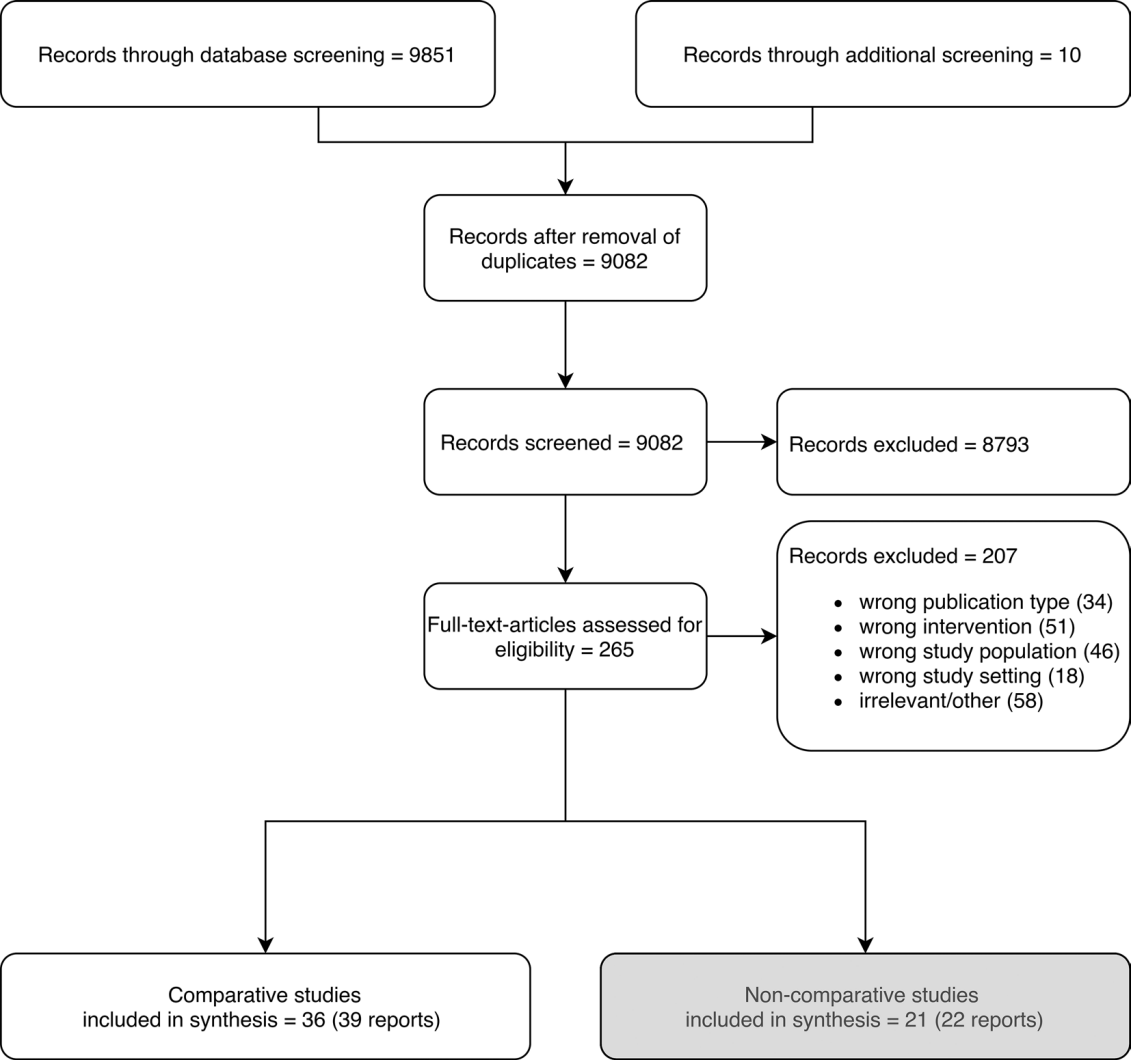
Flow of records through the screening process

We retrieved 9851 records from the database search. 10 additional records were obtained through citation screening and hand search. After removal of duplicates, 9081 records remained. 8739 records were excluded in title and abstract screening. For 265 records, the full text was accessed. A total of 39 articles reporting on 36 different datasets (including 5 additional studies showing an overlap in data but not using the same dataset) were included in the subset of comparative study designs. An overview of the flow of studies through the screening process can be seen in Fig. 2. For results of the critical appraisal of study quality see Additional file 2.

Cohen’s kappa was calculated as a measure of the agreement of the raters in the screening process. It indicated almost perfect agreement (0.85).

Publication dates ranged from 2004 to 2021. 8 articles report data from Australia [37–44], 7 from New Zealand [45–51], 3 Studies were conducted in Japan [52–54], the United States of America [55–57] and Norway [58–60], 2 reported on data gathered in different European countries [61, 62] and 1 study each came from Austria [63], Spain [64], France [65], Hong Kong [66], The Netherlands [67], Sweden [68] and Taiwan [69]. 5 articles did not specify the country [70–74]. They conveyed a total of 1791 participants ranging from 4 to 415 (mean: 49.75, median: 25) Further characteristics of the study population can be seen in Table 1. A brief overview on the study characteristics can be found in Table 2.

**Table 1:** Study characteristics

Article	Study type	Intervention	Comparison	Device
Bemelmans et al. 2015 [67]	quasi-experimental time series (ABAB -design)	Facilitation of daily care tasks	Care support intervention	Paro
Beuscher et al. 2017 [55]	Before/After	Facilitation of physical, social or cognitive activities in group and one-on-one		Nao
Bickmore et al. 2005 [56]	RCT	Facilitation of physical activities	Standard of care	FitTrack
Broadbent et al. 2016 [45]	CT	Availability for free interaction	Standard of care	Guide and Cafero
Broadbent et al. 2018 [46]	RCT	Facilitation of treatment adherence	Standard of care	iRobi
Chen et al. 2020 [75]	Before/After	Non-facilitated free interaction		Paro
D’Onofrio et al. 2019 [62]	Before/After	Facilitated interaction		MARIO
Demange et al. 2018 [65]	Before/After	Free interaction (non-participating supervision)		Paro
Fan et al. 2017 [70]	Before/After	One-on-one / triadic interaction		Nao (ROCARE)
Fischinger et al. 2016 [61]	Before/After	6 standardized care support operations performed by the robot		Hobbit
Gustafsson, Svanberg, and Müllersdorf 2015 [68]	Single-Case Study (A-B-A design)	Facilitation of physical, social or cognitive activities		Justo Cat
Jones et al. 2018 [37]	RCT	Individual non-factilitated interaction	Standard of care	Paro
Jøranson, Pedersen, Rokstad, and Ihlebaek 2016 [58]	RCT	Robot-assisted group acitivity	Standard of care	Paro
Jøranson, Pedersen, Rokstad, Aamodt, et al. 2015 [60]	RCT	Robot-assisted group acitivity	Standard of care	Paro
Jøranson et al. 2016 [59]	RCT	Robot-assisted group acitivity	Standard of care	Paro
Ke et al. 2020 [66]	RCT (ABAB-design)	Unstructured non-facilitated interaction	Standard of care	Kabochan
Khosla et al. 2021 [38]	Before/After	Interaction in home based care		Betty
Liang et al. 2017 [47]	RCT	Unstructured group sessions	Standard of care	Paro
Libin and Cohen-Mansfield 2004 [72]	Quasi-Experimental trial	Facilititated interactive sessions	Plush toy	NeCoRo
McGlynn et al. 2017 [57]	Before/After	Facilitated interaction	Plush toy	Paro
Mervin et al. 2018 [39]	RCT	Individual non-facilitated intervention	Plush toy and standard of care	Paro
Moyle et al. 2013 [40]	Randomised Crossover Study	Facilitation of physical, social or cognitive activities	Reading group intervention	Paro
Moyle et al. 2017 [41]	RCT	Individual non-facilitated sessions	Plush toy and standard of care	Paro
Petersen et al. 2016 [73]	RCT	Facilitated group intervention	Standard of care	Paro
Pripfl et al. 2016 [63]	Before/After	Free interaction in home care		Hobbit
Pu et al. 2020a [42]	RCT	Individual non-facilitated sessions	Standard of care	Paro
Pu et al. 2020b [43]	RCT	Individual non-facilitated sessions	Standard of care	Paro
Pu et al. 2021 [44]	RCT	Individual non-facilitated sessions	Standard of care	Paro
Robinson et al. 2013a [48]	Quasi-Experimental trial	Facilitated interaction with Paro	Facilitated interaction with Guide	Paro and Guide
Robinson et al. 2013b [49]	RCT	Robot-assisted discussion groups	Standard of care	Paro
Robinson, MacDonald, and Broadbent 2015 [50]	Quasi-Experimental (A-B-A design)	Facilitated interaction		Paro
Shibata et al. 2004 [71]	Before/After	Availability for free interaction	Availability for free interaction with placebo Paro (reduced functions	Paro
Valenti-Soler et al. 2015 [64]	RCT (2 phases)	Phase1/2: Paro and Nao in standardized occupational therapy sessions	Phase1: standard of care Phase 2: Dog therapy	Paro and Nao
Stafford et al. 2014 [51]	Before/After	Availability for facilitated interaction /individual sessions		Charlie
Sung et al. 2015 [69]	Before/After	Robot-assisted group therapy		Paro
Tamura et al. 2004 [52]	Quasi experimental trial	Robot-assisted occupational therapy	Toy	AIBO
Wada et al. 2005 [53]	Time series	Group interaction		Paro
Wada et al. 2006 [74]	Time series	Group interaction		Paro
Wada and Shibata 2008 [54]	Before/After	Availability for free interaction		Paro

**Table 2:** Study population characteristics

Article	n =	Investigated setting	Age in years	Health Characteristics
Bemelmans et al. 2015 [67]	71	Psychogeriatric care	n.a.	First stages of dementia
Beuscher et al. 2017 [55]	19	Living lab setting	range: 66 to 94	Normal cognitive abilities or mild stages of dementia
Bickmore et al. 2005 [56]	21	Home care	range: 63 to 85 mean: 74.0	No significant cognitive impairments; in need for physical exercise
Broadbent et al. 2016 [45]	52	Retirement village	range: 66 to 97 average: 85.	Normal cognitive abilities or cognitive impairment
Broadbent et al. 2018 [46]	60	Home care in recovery after COPD related hospital admission	mean (ctrl): 69.10; mean (interv.):70.57	Diagnosed COPD and COPD related admission, living alone or with spouse
Chen et al. 2020 [75]	20	Long term care	range: 65 to 93 mean: 81.1	Score > 6 in geriatric depression scale. 75% with diagnosed depression
D’Onofrio et al. 2019 [62]	38	Care setting	range: 55 to 93 mean: 77.08	Diagnosed with dementia
Demange et al. 2018 [65]	17	Geriatric Hospital	range: 77 to 95 mean: 83.0	Major neurocognitive disorders (agitation, depression)
Fan et al. 2017 [70]	25	Home care	range: 66 to 94	No cognitive impairments or diagnosed dementia
Fischinger et al. 2016 [61]	49	Living lab setting	> 70	Various grades of impairment in vision, hearing or mobility
Gustafsson, Svanberg, and Müllersdorf 2015 [68]	4	Care Home	range: 82 to 90	Late stage dementia
Jones et al. 2018 [37]	138	Care Home	mean: 84.0	Diagnosed dementia
Jøranson, Pedersen, Rokstad, and Ihlebaek 2016 [58]	27	Care Home	range: 62 to 95	Moderate to severe forms of dementia
Jøranson, Pedersen, Rokstad, Aamodt, et al. 2015 [60]	30	Care Home	range: 62 to 92 mean: 84.7	Moderate to severe forms of dementia
Jøranson et al. 2016 [59]	60	Care Home	range: 62 to 95	Moderate to severe forms of dementia
Ke et al. 2020 [66]	103	Care Home	mean: 87.2	Diagnosed dementia
Khosla et al. 2021 [38]	10 (in dyads of caregiver and care receiver)	Home care	range: 75 to 85	Care receiver with dementia
Liang et al. 2017 [47]	60 (in dyads of caregiver and care receiver)	Day care center and home care	range: 67 to 98	Diagnosed with dementia
Libin and Cohen-Mansfield 2004 [72]	9	Care Home	range: 83 to 98	Severe cognitive decline
McGlynn et al. 2017 [57]	30	Living lab setting	range: 67 to 80	Older adults living alone
Mervin et al. 2018 [39]	415	Care home	mean: 84.4	Diagnosed with dementia
Moyle et al. 2013 [40]	18	Care home	average: 85.3	Mid to late stage dementia
Moyle et al. 2017 [41]	415	Care home	mean: 84.4	Diagnosed with dementia
Petersen et al. 2016 [73]	61	Dementia care unit	average: 83.4	Mild to moderate forms of dementia
Pripfl et al. 2016 [63]	7	Home care	> 75	Living alone
Pu et al. 2020a [42]	43	Long term care	mean: 86.0	Dementia and symptoms of chronic pain
Pu et al. 2020b [43]	43	Long term care	mean: 86.0	Dementia and symptoms of chronic pain
Pu et al. 2021 [44]	43	Long term care	mean: 86.0	Dementia and symptoms of chronic pain
Robinson et al. 2013a [48]	21 (in dyads with relatives)	Retirement village and hospital care dementia unit	range (care receivers): 71 to 93	n.a.
Robinson et al. 2013b [49]	40	Care home	range: 55 to 100	48% scored > 6 on the Abbreviated Mental Test
Robinson, MacDonald, and Broadbent 2015 [50]	21	Rest home and hospital care dementia unit	range: 71 to 95	n.a.
Shibata et al. 2004 [71]	23	Day care facility	average (ctrl): 85.5 average (interv): 84.4	n.a.
Valenti-Soler et al. 2015 [64]	211	Care home	range: 58 to 101	Moderate to severe forms of dementia
Stafford et al. 2014 [51]	25	Retirement village	range: 78 to 95	n.a.
Sung et al. 2015 [69]	16	Care home	average: 77.25	Severe level of dependency.
Tamura et al. 2004 [52]	13	Geriatric care facility	average: 84	Severe dementia
Wada et al. 2005 [53]	8	Healthcare facility	n.a.	Most subjects with dementia
Wada et al. 2006 [74]	14	Healthcare facility	range: 77 to 98	Most subjects with dementia
Wada and Shibata 2008 [54]	12	Care facility	range: 67 to 89	Mini mental state examination scores between 15 and 23

In the complete dataset (comparative and non-comparative studies), 20 out of 58 publications referenced, highlighted or considered all three steps of the double-benefit argument. Within the subset which is reported here 10 studies made these references [47, 55–57, 61, 63, 66, 68, 70, 71]. In what follows we will present the results in line with our research question grouped by devices and evidence relevant to each of the two claims of the double-benefit argument.

### Paro

The most commonly investigated device was Paro. Paro is a companion robot specifically designed to stimulate interaction and provide comfort to its users. It mimics the outward appearance of a baby seal and is equipped with sensors for touch and sound. It can move its head, flippers and tail and makes seal-like noises such as cooing and whistling. It also includes a basic model of emotions to display states such as happiness, anger or sleepiness and can learn to respond to acoustic stimuli (e.g. a name). With regard to Paro 7 studies found significant effects relevant to the well-being claim [40, 47, 49, 58, 59, 65, 75]. 3 studies reported that the use of Paro has stabilizing or positive effects on the Quality of Life of study participants which seem to develop over a longer period of time ( 7 to 10 weeks) [41, 58, 75]. Chen et al. and Robinson et al. both found positive effects on perceived feelings of loneliness (which is also one of the subscales to measure the overall quality of life) [49, 75]. 3 studies investigated the effects of Paro on the perceived overall mood as part of emotional well-being and found that its use can lead to an improvement [47, 59, 65].

A greater portion (13) of the studies investigating the effects of Paro were concerned with the potential mitigation of negative effects. 2 studies found evidence that the use of Paro can decrease the need for medication to mitigate psychiatric symptoms such as anxiety or agitation in elderly populations. Joranson et al. reported a significant decrease in psychotropic medication for participants with severe forms of dementia, which was slightly smaller in groups with less severe forms [58]. Petersen reported on a reduction of medication for mitigation of behavioral symptoms and pain [73]. Pu et al. reported similar results with regard to pain medication [42, 43]. Reducing prescriptions will almost certainly result in fewer side effects and hence might have additional positive effects on well-being. Jones et al. and Joranson et al. additionally report having found a decrease in agitated behavior in persons using Paro [37, 60]. This is in line with a general tendency to decreased symptoms of depression, including anxiety or sadness, which was reported in 5 studies [40, 41, 53, 60, 64, 73, 74]. In addition, it was reported a positive effect on cardiovascular parameters such as heart rate, blood pressure, and pulse oximetry that occurred after interacting with Paro [47, 49, 73].

Despite these overall positive effects on emotional and physical well-being, several studies note that these results are dependent on the characteristics of the study population, the type of intervention the device was used with as well as several other factors. For example, three studies note that positive effects in social behavior, increased social interactions or increased active engagement of their participants occur with group interventions [47, 54, 69] while they are not reported in similar settings with individually facilitated or individual non-facilitated interventions [57]. As noted above, Joransson et al. note differences between different forms of dementia [58]. Additionally, Demange et al. report that effects on agitated persons using Paro were reduced more effectively than symptoms of depressed persons [65]. 2 Studies show that persons with milder forms of dementia were more likely to engage with the Device [47, 59] while Robinson et al. indicate that positive effects on cardiovascular parameters may depend on the level of engagement of participants [50]. Jones et al. conclude that a higher cognitive level of function leads to significantly better results. Given some trends in their data turning towards negative effects regarding well-being of participants, they suggest restricting the use to persons with low to moderate agitation symptoms while it might not be suitable for dementia patients [37]. In line with these results, Moyle et al. report increased wandering tendencies in persons with severe dementia compared to standard of care [40].

Evidence on the efficiency claim is much weaker in studies investigating the effects of Paro. Moyle et al. found that Paro was more effective than the usual care intervention it was compared to. This applies especially with regard to positive effects on mood and improving pleasure in dementia patients [41]. In addition, it was found that Paro might effectively mitigate symptoms of anxiety while effects on other symptoms of depression were minimal compared to usual care [40, 41, 71]. 2 Studies report Paro to be comparable to animal therapy with regard to its cardiovascular effects [49, 50]. Compared to a plush toy, participants were significantly more engaged verbally and visually with Paro [41]. However, 2 studies showed that there are only very few differences between using Paro and using a plush toy or a Placebo Paro (the same device with functions deactivated) [41, 71]. In addition, one study investigated the differences between Paro and another device (Nao) and found no differences except for different engagement patterns [48]. Surprisingly, Cost-effectiveness in achieving these and other effects was only investigated in one study [39]. It found that Paro and a plush toy might be more cost-effective than the standard of care intervention. However, Paro was far less cost-effective than a plush version.

### Nao

Nao is an upright robot with humanoid features. It has a freely programmable control unit that enables different scenarios of use. It is equipped with cameras, microphones and speakers and has voice recognition, face recognition and object tracking to enable interaction. The effects of Nao were investigated in 3 Studies. Beuscher et al. and Fan et al. investigated the effects on the acceptance of Nao [55, 70]. Both studies showed significant positive effects after interacting with the devices except for items on the subscales for anxiety towards SATs. Similar to Paro, Valenti-Soler et al. show that Nao can have positive effects on emotional well-being and contributes to the decrease of symptoms of depression as well as a decrease in the severity of neuropsychiatric symptoms such as the occurrence of delusions, apathy or irritability [64]. No data was found with regard to the efficiency claim.

### Guide and Hobbit

Hobbit is a multifunctional care robot with anthropomorphized features (e.g. head and face) that is designed to support aging in place. It is mounted on a mobile platform to move within a person’s living space. Its main goal is to provide fall protection through object detection and picking up objects from the floor, as well as through patrolling and handling emergencies [61]. In addition, it provides cognitive assistance and entertainment functions. It can be controlled via touch screen or voice command. Guide is an about 1.6 m tall care robot with anthropomorphic features. It interacts by speaking or through its touch screen. Guide comes with a programmable software platform that includes the ability to take vital signs, provide entertainment and brain fitness games as well as video-call capabilities [48].

Results on the effects of Guide and Hobbit were rather inconclusive. In comparison to other devices, Broadbent et al. report that using Guide in their study was not connected to any major benefits in well-being [45]. This includes inconclusive results in a decrease in depressive symptoms and a potential increase in quality of life. As mentioned before, Guide seems to induce less engagement in its users than Paro [48]. Hobbit on the other hand was investigated in 2 studies and is reported to be connected to an increase in an overall positive attitude of users. However, exceptions occur in certain subscales such as in negative attitudes towards the social influence of the device or negative attitudes towards interaction [61, 63].

### Non-aggregated results

The remaining studies presented results on various devices. Given this variety, we did not deem it suitable to further synthesize these studies. A brief overview of the studies and main results is presented in Table 3.

**Table 3:** Non-aggregated results

Article	Device	Results
Bickmore et al. 2005 [56]	FitTrack	Significant increase in mean steps per week walked; Significant increase compared to standard of care. No significant differences in well-being or loneliness.
Broadbent et al. 2016 [45]	Cafero	No significant differences in depression, quality of life, mobility activities of daily living, or behavioral scores compared to intervention with Guide
Broadbent et al. 2018 [46]	iRobi	Increased medication and therapy adherence. Net cost benefit compared to the control group.
D’Onofrio et al. 2019 [62]	Mario	No significant differences in affective status or Quality of Life compared to pre-intervention. Significant increase in resilience compared to pre-intervention.
Gustafsson, Svanberg, and Müllersdorf 2015 [68]	JustoCat	Indication of an increase in Quality of Life and decrease of symptoms of agitation as compared to pre-intervention. Not statistically significant due to the method used.
Libin and Cohen-Mansfield 2004 [72]	NeCoRo	Decrease of symptoms of agitation, affect. No significant differences compared to plush toy. Positive increase in engagement, no differences compared to plush toy. Engagement related to cognitive impairment.
Stafford et al. 2014 [51]	Charlie	Computer knowledge and positive attitude towards robots is a predictor of robot uptake and leads to lesser attribution of mind agency in robots.
Tamura et al. 2004 [52]	AIBO	Increase in engagement in occupational therapy. No differences compared to toy dog.
Khosla et al. 2021 [38]	Betty	Increase in engagement and frequency and duration of interaction.

## Discussion

The double-benefit argument claims that SATs may provide opportunities to increase individual well-being and autonomy of their users. At the same time, SATs might be a means to mitigate increasingly scarce resources in healthcare as they present themselves as more efficient compared to standard care or other available alternatives yielding at least similar results. On grounds of ethical claims to avoid harm and maintain autonomy and well-being, it can be argued that research, implementation and use of SATs should be broadly considered. The validity of the double benefit argument is based on its two empirical claims, that is, SATs do benefit and do not harm their users and contribute to maintaining or increasing autonomy and well-being. Secondly, they do provide a more efficient means to the same end and, hence, contribute to mitigating scarce resources. From an ethical perspective, our review demonstrates that these claims should be handled with caution when used in ethical debates or to guide empirical research. To our understanding, the analysis of the state of empirical evidence, first, suggests a careful use of the well-being claim with regard to its generalizability and, secondly, does not lend support to the efficiency claim. In what follows, we will elaborate on these two findings in more depth.

### Generalizability of the well-being claim

Empirical evidence supporting the well-being claim is well documented at least for Paro. This applies especially with regard to positive outcomes in changes of mood, symptoms of agitation and a generally increased Quality of Life in study participants - all of which indicate a benefit either directly or indirectly to physical or emotional well-being as well as positive effects for the ability of self-determination. There is, however, additional evidence suggesting that this overall positive outcome does not occur in all population groups nor with all types of interventions. The work of Jones et al., for example, demonstrates significantly better results in persons with higher functional capacities [37] while others seem to confirm these findings. With some trends even turning towards negative outcomes in patients with dementia, study authors suggest restricting the use of Paro to persons with mild cognitive symptoms and agitated behavior and to exclude persons with more severe forms of dementia.

These results are important as they highlight the context-dependency of the well-being claim. The use of devices like Paro seems to lead to more positive results when used with a human component such as in group interventions or facilitated sessions with persons suffering from rather mild neuropsychiatric symptoms such as mild agitation. This does, however, not include the most commonly discussed scenarios of use, which, in line with the above-noted demographic projections, often include persons with more severe forms of dementia in need of more intensive care. In conclusion, these findings suggest that the well-being claim is not generalizable and does not apply to all populations and intervention types. Using it as a basis to support respective arguments, hence, requires context-specific validation. In addition, it underlines the need for more research investigating this context-dependency to allow for a more fine-grained evaluation of the potential benefits. This implies carefully reflecting on the selection of study populations. As Table 1 shows, the overwhelming majority of results refer to groups of people with significant limitations due to dementia.

With a view to the well-being claim and specific contexts, our results also raise questions in regard to potential negative effects. The indication of potentially negative outcomes raises concerns that settings may exist in which certain groups of persons might actually experience negative effects in care arrangements including SATs. From an ethical perspective, the data suggests to consider users’ capacities and competencies as well as relational aspects in connection to human components of interventions as important factors of ethically desirable scenarios of use. If this finding could be substantiated, it would be significant from an ethical perspective as it may require a more complex ethical calculus than the one suggested by the double-benefit argument.

### Limited support for increased efficiency

With regard to the efficiency claim, we found scarce empirical evidence in support. The results show that the use of SATs such as Paro can be comparable to animal therapy. In addition, two studies presented a weak correlation between the use of SATs and decreased use of medication compared to a usual care scenario. However, no major differences were found comparing, for example, Paro or Nao to a plush toy or a placebo device. Finally, cost-effectiveness as one of the most important measures to indicate any potential to achieve the same end with the same or fewer (financial) resources was only scarcely investigated. It was found that Paro was ineffective compared to a plush version. In addition, we found no evidence supporting any claim that care workers could actually be relieved from tasks or capacities gained through the use of SATs were used for other care work. This does not include a perceived subjective relief in care workers as potential results in this regard were not part of our dataset. From an ethical perspective, it needs to be highlighted that this significantly weakens the plausibility of the double-benefit argument. Combining these assessments with the above-noted tendency of positive results in group or facilitated interventions casts further doubt upon the efficiency claim. Both insights together suggest that the use of SATs may not necessarily lead to an increase in the efficiency of care interventions because, for example, care workers are relieved from certain tasks and can invest time and resources elsewhere. Rather, it can be hypothesized that a shift in tasks and responsibilities occurs with care workers acting in the role of mediators, supervisors, providers or controllers of the technical components of the intervention. This would be in line with known observations from other areas of the digitalization of healthcare, where it has been shown that the digital transformation leads to a fundamental change in healthcare practices leading to new roles and responsibilities [76, 77]. In sum, we suggest that the validity of the efficiency claim cannot be established. The burden of proof consequently remains with the proponents of this argument.

While these considerations on the grounds of the empirical evidence mostly apply to Paro, it is important to note that studies using different devices are still rare and provide even less evidence for said claims. Furthermore, the critical appraisal we conducted shows a mixed study quality which tends to decline with regard to further devices. A general problem is to be noted with regard to the question of representative target populations. Like all studies that investigate the interaction between humans and technology in healthcare, the studies in our dataset are dependent on the willingness of the participants to engage with the devices and thus tend to include participants with a positive attitude and exclude participants with a negative attitude towards this kind of technology. As this attitude also affects how persons interact with SATs, the selection of participants entails a strong risk of introducing a selection bias, which we did not find sufficiently addressed in many studies.

## Conclusion

We conclude from these observations that current research does not support the double-benefit argument univocally as a background against which ethical debates should be situated or empirical research should be conducted. Notwithstanding this conclusion, we highlight that almost one-third of the studies in our dataset included references to the double benefit argument. Although it has been intensively criticized in the philosophical debate and although our results might cast doubt on its current plausibility it is - and probably continues to be - a strong driving force in respective research frameworks. It is well known, however, that value-laden assumptions such as those coming with the argument and which are either implicitly or explicitly made within research frameworks often significantly shape respective practices and influence developmental processes, research goals and foci of investigation. From an ethical perspective, this raises the question of whether and to what extent a more careful reflection and framing of such research with regard to its goals and investigated outcomes might be necessary.

We do not deny, however, that this conclusion has its limitations. For the first, we do not want to claim, that the double-benefit argument is logically fundamentally flawed, inconsistent or outright wrong. On contrary, if proven valid for a certain device or under certain circumstances, it would, to our understanding, provide strong reason to think about the further use of SATs as a prima facie obligation. A prima facie obligation can be said to exist when there is compelling reason for a certain act that holds until it is overwritten by stronger moral considerations. If it could be shown that a certain device is more efficient than existing alternatives and that it is beneficial to its users and does not threaten their autonomy, there would indeed be a case to make for an obligation. This obligation could only be outweighed by additional ethical considerations (e.g. by considering the value of human contact or the principled impermissibility of deceptions or other fundamental arguments). The burden of proof would, then, reside with the opponents of such prima facie obligation. However, as a mixed moral judgment based on normative assumptions and empirical claims, we do not find it sufficiently supported in the current state of knowledge to consider the threshold for compelling reason met. This does not preclude a change given the development of devices or insights from new research. In addition, our results do not render further ethical considerations in regard to the double benefit argument obsolete, but merely show that a more detailed consideration of the assumptions of the argument and a more reflected use is necessary. Secondly, it has to be added that the generalizability of our findings might be limited due to the dataset and especially due to the variety of studies, study types and interventions. Given the difference in, for example, used scales in measuring respective outcomes, we did not conduct a statistical analysis of the data. Pooling the available data might yield additional insights that could be helpful in developing future research directions or developing a more detailed picture. Finally, it has to be kept in mind that the analyzed data emerges from a variety of different countries and cultural contexts. Results may covary with these backgrounds limiting their comparability.

### Electronic supplementary material

Below is the link to the electronic supplementary material.

Additional file 1: PRISMA-checklist.pdf; Checklist of reported items.

Additional file 2: Critical Appraisal.pdf; Critical Appraisal of the methodological quality of included studies.

## Data Availability

The datasets used and analysed during the current study are available from the corresponding author on reasonable request.

## List of abbreviations

SAT: Socially assistive devices
MMAT: Mixed-Methods Appraisal Tool
PRISMA: Preferred Reporting Items for Systematic Reviews and Meta-Analyses
