# Supplementary material for: Increasing efficiency and well-being? a systematic review of the empirical claims of the double-benefit argument in socially assistive devices
Source: BMC Med Ethics. 2023 Nov 30;24:106. doi: 10.1186/s12910-023-00984-z (PMC10687833; doi:10.1186/s12910-023-00984-z)
Supplement: Supplementary file 2 — Additional file 2: Critical Appraisal.pdf; Critical Appraisal of the methodological quality of included studies. [file 12910_2023_984_MOESM2_ESM.pdf]

Additional file 2 to: Increasing efficiency and well-being? A systematic review of the empirical claims of the double benefit argument in socially assistive devices

Joschka Haltaufderheide (1,2,3), Annika Lucht (2), Christoph Strünck (4) & Jochen Vollmann (2)

(1) Junior Professorship of Medical Ethics with a Focus on Digitization. Joint Faculty for Health Sciences Brandenburg, University of Potsdam, Germany

(2) Institute for Medical Ethics and History of Medicine, Ruhr-University Bochum, Germany

(3) Corresponding Author

(4) School of Life Sciences, University of Siegen, Germany, Institute of Gerontology at Technical University Dortmund, Germany

Critical Appraisal of study quality

		Bemelsmans et al. 2015	Beuscher et al. 2017	Bickmore et al. 2005	Broadbent et al. 2016	Broadbent et al. 2018	Chen et al. 2020	Chen (Ke) et al. 2020	D'Onofrio et al. 2019	Demange et al. 2018	Fan et al. 2017	Fischinger et al. 2016	Gustafsson et al. 2015	Jones et al. 2018	Jøranson et al. 2015	Jøranson et al. 2016 a	Jøranson et al. 2016 b	Khosla et al. 2019	Liang et al. 2017
**Category of Study Designs**	**Methodological Quality Criteria**																		
Questions for all types	Are there clear research questions?	y	y	y	y	y	y	y	y	y	y	y	y	y	y	y	y	y	y
	Do the collected data allow to address the research questions?	y	y	y	y	y	y	y	y	y	y	y	y	y	y	y	y	y	y
**Quantitative randomized controlled trials**																			
	Is randomization appropriately performed?			?		y		y						y	y	y	y		y
	Are the groups comparable at baseline?			n		y		y						y	y	y	y		y
	Are there complete outcome data?			y		y		y						y	y	y	y		y
	Are outcome assessors blinded to the intervention provided?			?		?		y						y	n	n	n		n
	Did the participants adhere to the assigned intervention?			y		y		y						y	y	y	y		y
**Quantitative non-randomized**																			
	Are the participants representative of the target population?	n	n		?		n		?	n	?	y	n					?	
	Are measurements appropriate regarding both the outcome and intervention (or exposure)?	y	y		y		y		y	y	y	y	y					y	
	Are there complete outcome data?	y	y		y		y		y	y	y	y	y					y	
	Are the confounders accounted for in the design and analysis?	?	?		y		y		?	y	?	?	?					?	
	During the study period, is the intervention administered (or exposure occurred) as intended?	y	y		y		y		y	y	y	y	y					y	
**Mixed methods**																			
	Is there an adequate rationale for using a mixed methods design to address the research question?						y						y					y	
	Are the different components of the study effectively integrated to answer the research question?						y						y					y	
	Are the outputs of the integration of qualitative and quantitative components adequately interpreted?						y						y					y	
	Are divergences and inconsistencies between quantitative and qualitative results adequately addressed?						y						y					?	
	Do the different components of the study adhere to the quality criteria of each tradition of the methods involved?																		

		Libin et al. 2004	McGlynn et al. 2017	Mervin et al. 2018	Moyle et al. 2013	Moyle et al. 2017	Petersen et al. 2017	Pripfl et al. 2016	Pu et al. 2020 a	Pu et al. 2020 b	Pu et al. 2021	Robinson et al. 2013a	Robinson et al. 2013b	Robinson et al. 2015	Shibata et al. 2004	Soler et al. 2015	Stafford et al. 2014	Sung et al. 2014	Tamura et al. 2004	Wada et al. 2005	Wada et al. 2006	Wada/Shibata 2008
**Category of Study Designs**	**Methodological Quality Criteria**																					
Questions for all types	Are there clear research questions?	y	y	y	y	y	y	y	y	y	y	y	y	y	y	y		y	y	y	y	y
	Do the collected data allow to address the research questions?	y	y	y	y	y	y	y	y	y	y	y	y	y	y	y		y	y	y	y	y
**Quantitative randomized controlled trials**																						
	Is randomization appropriately performed?			y	y	y	y		y	y	y		y			y						
	Are the groups comparable at baseline?			y	y	y	y		y	y	n		y			y						
	Are there complete outcome data?			y	n	y	y		y	n	y		y			y						
	Are outcome assessors blinded to the intervention provided?			y	n	y	n		n	n	n		n			y						
	Did the participants adhere to the assigned intervention?			y	y	y	y		y	y	y		y			y						
**Quantitative non-randomized**																						
	Are the participants representative of the target population?	n	n					y				?		?	n		n	n	n	n	n	n
	Are measurements appropriate regarding both the outcome and intervention (or exposure)?	y	y					y				y		y	y		y	y	y	y	y	y
	Are there complete outcome data?	y	n					n				y		y	y		y	y	y	n	?	y
	Are the confounders accounted for in the design and analysis?	?	?					?				n		y	?		y	y	?	?	?	?
	During the study period, is the intervention administered (or exposure occurred) as intended?	y	y					?				n		y	?		y	y	n	?	?	y
**Mixed methods**																						
	Is there an adequate rationale for using a mixed methods design to address the research question?		y					y				y										
	Are the different components of the study effectively integrated to answer the research question?		y					n				y										
	Are the outputs of the integration of qualitative and quantitative components adequately interpreted?		y					y				y										
	Are divergences and inconsistencies between quantitative and qualitative results adequately addressed?		y					y				y										
	Do the different components of the study adhere to the quality criteria of each tradition of the methods involved?		y					n				y										
